# A systematic review of the effects of CYP2D6 phenotypes on risperidone treatment in children and adolescents

**DOI:** 10.1186/s13034-018-0243-2

**Published:** 2018-07-16

**Authors:** Thomas Dodsworth, David D. Kim, Ric M. Procyshyn, Colin J. Ross, William G. Honer, Alasdair M. Barr

**Affiliations:** 1 Department of Pharmacology, University of British Columbia, 2176 Health Sciences Mall, Vancouver, BC V6T 1Z3 Canada; 2 Department of Psychiatry, University of British Columbia, Vancouver, BC Canada; 3 Faculty of Pharmaceutical Sciences, University of British Columbia, Vancouver, BC Canada

**Keywords:** 2D6, Adolescents, Antipsychotic, Cytochrome P450, Pharmacogenomics, Psychopharmacology, Risperidone

## Abstract

The second generation antipsychotic drug risperidone is widely used in the field of child and adolescent psychiatry to treat conditions associated with disruptive behavior, aggression and irritability, such as autism spectrum disorders. While risperidone can provide symptomatic relief for many patients, there is considerable individual variability in the therapeutic response and side-effect profile of the medication. One well established biological factor that contributes to these individual differences is genetic variation in the cytochrome P450 enzyme 2D6. The 2D6 enzyme metabolizes risperidone and therefore affects drug levels and dosing. In the present review, we summarize the current literature on 2D6 variants and their effects on risperidone responses, specifically in children and adolescents. Relevant articles were identified through systematic review, and after irrelevant articles were discarded, ten studies were included in the review. Most prospective studies were well controlled, but often did not have a large enough sample size to make robust statements about rarer variants, including those categorized as ultra-rapid and poor metabolizers. Individual studies demonstrated a role for different genetic variants in risperidone drug efficacy, pharmacokinetics, hyperprolactinemia, weight gain, extrapyramidal symptoms and drug–drug interactions. Where studies overlapped in measurements, there was typically a consensus between results. These findings indicate that the value of 2D6 genotyping in the youth population treated with risperidone requires further study, in particular with the less common variants.

## Background

Risperidone is a second generation (“atypical”) antipsychotic drug used for the treatment of multiple psychiatric disorders, including schizophrenia, bipolar disorder and symptoms associated with autism spectrum disorder (ASD) (FDA Label 2009). It is used to treat both children and adults. In children and adolescents, risperidone was the second most commonly used antipsychotic drug in the United States by 2006 and continues to be widely used in various psychiatric disorders prevalent in pediatric populations, including bipolar disorder, schizophrenia, attention deficit hyperactivity disorder, and ASD (e.g., symptoms of irritability) [1–5]. Side effects associated with risperidone treatment include weight gain, glucose dysregulation, hyperprolactinemia, and extrapyramidal symptoms [6, 7] as well as less common but severe reactions including cardiovascular effects [8] and neuroleptic malignant syndrome [9]. Children and adolescents are especially prone to adverse side effects and variations in therapeutic outcome associated with risperidone treatment [6, 10]. Variation in drug treatment outcomes between youth and adults is a well-characterized phenomenon in pharmacological research. This may reflect biological differences, such as in organ and tissue proportions, body fluid distribution, and protein composition of serum, all of which are factors that may contribute to such variations [6, 11]. As with all antipsychotic drugs, risperidone’s pharmacodynamics and pharmacokinetics are influenced by multiple factors including age, sex, ethnicity, nutritional status, smoking and alcohol use [12]. The present review considers the importance of pharmacogenomic factors, with a specific focus on one confounding factor that significantly affects risperidone treatment outcome: CYP2D6 metabolic phenotype. The word “outcome” is intentionally used broadly to include such factors as efficacy, pharmacokinetics, prevalence of adverse side effects, and effects of concomitant drug use.

CYP2D6 is a liver enzyme involved in the metabolism of approximately 25% of drugs in use today [13]. The gene for CYP2D6 is highly polymorphic: there are > 100 allelic variants for the 2D6 gene, including complete deletion and duplications of the gene [14]. Deviations in the number and type of allelic variants as well as gene copy number yield four CYP2D6-predicted metabolic phenotypes: ultra-rapid metabolizer (UM), extensive metabolizer (EM), intermediate metabolizer (IM), and poor metabolizer (PM) [12, 15]. Ultra-rapid metabolizers have CYP2D6 gene duplication in the absence of any inactive alleles. Extensive metabolizers have two functional wild-type CYP2D6 alleles. Intermediate metabolizers have two decreased-activity alleles or one decreased activity allele and one inactive allele or one active allele and one inactive allele. Poor metabolizers have two inactive alleles. In general, while the EM phenotype consists the majority of the general population (approximately 72–88%), occurrences of PM and UM phenotypes are less common at approximately 1–20 and 1–10%, respectively [16], and vary significantly according to ethnicity: for example, the PM phenotype is found in 7% of Caucasians but only 1% of Asians, while the UM phenotype is found in 2% of Caucasians and up to 25% of some Ethiopian ethnic groups [11]. As risperidone is primarily metabolized by CYP2D6 [17], which can therefore affect drug levels in both youth [18] and adults [19], different phenotypes may have significant clinical importance with regards to adverse side effects and drug effectiveness. While the importance of CYP2D6 genotype continues to be discussed for adult patients [16], there is little systematic information available for children and adolescents, who exhibit a wide range of risperidone drug levels [20].

Risperidone is converted by the CYP2D6 enzyme [21, 22] to its main metabolite, 9-hydroxyrisperidone, which is a pharmacologically active metabolite considered equipotent to the parent drug (marketed in its own right as the antipsychotic paliperidone). CYP3A4, albeit to a lesser extent, also contributes to the metabolism of risperidone to 9-hydroxy-risperidone. Evidence suggests that they have similar receptor affinities and efficacies, and both are primarily excreted in urine [23]. Since the conversion of risperidone to 9-hydroxyrisperidone is mediated by CYP2D6, the ratio of the two compounds (risperidone/9-hydroxyrisperidone ratio) in serum after allowing time for metabolism is correlated to CYP2D6 metabolic phenotype [21]. Poor metabolizers typically have a greater proportion of risperidone (less metabolic conversion) as CYP2D6 activity is low, while extensive and ultra-rapid metabolizers have a greater proportion of 9-hydroxyrisperidone [24]. A change in the ratio of the drug and its metabolite is postulated to be the primary mechanism by which CYP2D6 metabolic phenotypes produce variability in risperidone treatment outcomes [13, 24].

This systematic review investigates how CYP2D6 metabolic phenotypes affect outcomes of risperidone treatment (i.e., efficacy, pharmacokinetics, prevalence of adverse side effects, and effects of concomitant drug use) in children and adolescents. The review primarily evaluates the clinical importance of its findings and considers the overall value of CYP2D6 pharmacogenomic testing for young risperidone users.

## Methods

An OVID (July 2017) electronic search of the MEDLINE and EMBASE databases was performed to find studies that examined the effects of CYP2D6 metabolic phenotypes on risperidone treatment outcomes (i.e., efficacy, pharmacokinetics, prevalence of adverse side effects, and effects of concomitant drug use) in children and adolescents, using the following search strategy: “Cytochrome P450 Enzyme System” or “CYP2D6” and “Antidepressive Agents” or “Antipsychotic Agents” or “antidepress*” or “antipsychotic*”. Results were limited to English language and studies in humans and “all child (0–18 years)” age range. The search generated 228 results. 193 results were eliminated for irrelevancy; most were eliminated for not meeting the children and adolescents age limit because most studies were tagged with all age groups including children despite studying only adult subjects. Studies that included subjects over age 18 were included if the median or mean age of the study population was less than 18. Of the 35 relevant results, 11 were focused primarily on risperidone and CYP2D6. The scope of the literature review was subsequently narrowed to focus on this single drug and enzyme. Two risperidone studies were eliminated for irrelevancy after in-depth review, and one was added from scanning references lists. In total, 10 studies were included in the literature review. The search also yielded several relevant articles used for background information and discussion purposes.

## Results and discussion

### General characteristics of studies

A summary of the literature review is presented in Table 1.

**Table 1:** Summary of literature review on CYP2D6 genetic polymorphisms and risperidone use in children and adolescents

Authors	Dose and length of time on risperidone, mean (SD)	Population	CYP2D6-predicted phenotypes	Outcomes measured	Select results, mean (SD)	Limitations
Sukasem et al. [27]	1 (0.93) mg/day for 46.06 months	147 subjects All Thai ethnicity Age range 3–19, mean age 9.52 127 (86%) males All diagnosed with ASD	UM = none EM = 73 (50%) IM = 74 (50%) PM = none	Serum prolactin concentration Hyperprolactinemia defined as prolactin levels > 97.5‰, normalized for age and sex	Serum prolactin concentration (ng/mL)^a^: UM = no data EM = 16.90 (9.53–25.50) IM = 16.55 (11.28–24.08) PM = no data No significant difference in serum prolactin concentrations between phenotypes. No significant difference in presence or absence of hyper-prolactinemia between phenotypes	No UM or PM subjects
Vanwong et al. [18]	0.5 (0.50–1.00)^a^ mg/day for at least 4 weeks	84 subjects All Thai ethnicity Age rage 3–20, median age 10 (6.83–11.55)^a^ 75 (89.29%) males All diagnosed with ASD	UM = 4 (5%) EM = 46 (55%) IM = 33 (40%) PM = none 1 subject excluded from phenotyping	Serum risperidone concentration and risperidone/9-hydroxyrisperidone ratio	Serum risperidone concentration (ng/mL)^a^: UM = 0.0 (0.00–5.18) EM = 0.43 (0.00–1.53) IM = 1.85 (0.67–4.25) PM = no data Concentration in IM phenotype was significantly greater than EM but not UM. Risperidone/9-hydroxy-risperidone ratio in IM phenotype was significantly greater than both EM and UM	No PM subjects. Mean/median length of time on risperidone not reported
dos Santos Júnior et al. [34]	2.2 (1.3) mg/day in hyperprolactinemia group and 1.9 (1.2) mg/day in non-hyperprolactinemia group for 23.4 (28.6) months in hyperprolactinemia group and 30.9 (23.9) months in non-hyperprolactinemia group	120 subjects Varying ethnicities Age range 8–20, mean age 13.0 (3.1), median age 13 98 (82%) males Diagnosed with various psychiatric disorders 197 subjects not taking risperidone included as controls	UM = none EM = 76 (63%) IM = 37 (31%) PM = 7 (6%)	Serum prolactin concentration Hyperprolactinemia defined as > 20 mg/dL in males and > 25 mg/dL in females in absence of hypothyroidism. Patients grouped into “case” (hyperprolactinemia) and “control” (no hyperprolactinemia)	Number of cases/number of controls: UM = no data EM = 51/26 IM = 24/12 PM = 4/3 No significant difference in presence or absence of hyperprolactinemia between phenotypes	No UM subjects
Youngster et al. [30]	1.0 mg/day^a^ in IM/EM group; 0.65 mg/day^a^ in PM group; 1.25 mg/day^a^ in UM group for minimum 3 months, median duration 6 months	40 subjects Race/ethnicity data not given Age range 3–18, median age 7 34 (85%) males All diagnosed with ASD	UM = 2 (5%) EM or IM = 36 (90%) PM = 2 (5%)	Reported ADRs: weight gain and neurological extrapyramidal symptoms Clinical response: improvements in disruptive behaviour Serum prolactin concentration Serum risperidone and 9-hydroxyrisperidone concentrations	Number of subjects who reported ADRs: UM = 0 EM or IM = 9 PM = 2 Clinical response: UM = 0 EM or IM = 24 PM = 2 Serum prolactin concentration (mg/L)^a^: UM = 18.3 (17.2–19.4) EM or IM = 20.2 (6.5–65.6) PM = 50.3 (48.4–52.2) Serum risperidone concentration (ng/mL)^a^: UM = 0.75 (0.5–1.0) EM or IM = 1.0 (0–47) PM = 9.0 (6–12) All PM and UM patients diagnosed with hyper-prolactinemia Serum risperidone concentration significantly greater in PM phenotype	Too few UM and PM subjects. Hyperprolactinemia not defined
Roke et al. [11]	1.6 (1.0) mg/day for 53.3 (28.7) months	47 subjects 46 (98%) Caucasian Age range 10–19, mean age 14.7 (2.1) 47 (100%) males 45 (96%) diagnosed with ASD, 2 (4%) diagnosed with DBD	UM = 2 (4%) EM = 25 (54%) IM = 17 (37%) PM = 2 (4%)	Serum prolactin concentration Hyperprolactinemia defined as prolactin levels > 97.5%, normalized for age and sex	Serum prolactin concentration (ng/mL): UM = 6.8 (6) EM = 19.8 (17) IM = 18.4 (17) PM = 49 (0) No significant difference in serum prolactin concentrations between EM and IM phenotypes. Too few subjects for statistical testing in UM and EM. All PM patients met criteria for hyperprolactinemia diagnosis	Too few UM and PM subjects for statistical tests. No suggested mechanism for results, unlike Troost et al. who had contradictory findings
Sherwin et al. [12]	2.0 (1.5) mg/day	45 subjects but only 28 (62%) underwent CYP2D6 genotyping 42 (93%) Caucasian Age range 2–21, mean age 9.6 (3.7) 40 (89%) males Most diagnosed with ASD	UM = none EM = 15 (54%) IM = 6 (21%) PM = 7 (25%)	Relative clearance of risperidone CL/F (litres/hour)	Relative clearance of risperidone CL/F (litres/hour): UM = no data EM = 37.4 IM = 29.2 PM = 9.4 Decreased clearance significantly associated with decreased CYP2D6	No UM subjects. Length of time on risperidone not reported
Calarge et al. [36]	0.03 (0.03) mg/kg/day for at least 6 months	107 subjects 88 Caucasian, 10 African American, 5 Hispanic, 4 Other Age range 7–17, mean age 11.4 (2.8) 98 (92%) males Diagnosed with various psychiatric disorders	CYP2D6-predicted phenotype not determined. Instead, patients grouped according to concomitant use of CYP2D6 inhibiting drugs^b^ Group 0 = 51 (48%) Group 1 = 13 (12%) Group 2 = 10 (9%) Group 3 = 33 (31%)	Serum risperidone and 9-hydroxyrisperidone concentrations	Concentration of risperidone: Group 3 > Group 0 and Group 1 > Group 0 Concentration of active moiety (risperidone + 9-hydroxyrisperidone): Group 3 > Group 0. All other differences were insignificant. Full numerical data not given, only bar graph	Patients were not genotyped, but implications for CYP2D6-predicted phenotypes combined with CYP2D6 inhibitors are explained
Correia et al. [29]	1.0, 2.0 or 3.0 mg/day based on weight for 12 months	45 subjects 44 (98%) Caucasian Age range 3–21, mean age 8.67 (4.30) 34 (76%) males All diagnosed with ASD	UM = 8 (18%) EM = 24 (53%) IM = 12 (27%) PM = 1 (2%)	Autism Treatment Evaluation Checklist (ATEC) score (for efficacy) BMI Waist circumference. Serum prolactin concentration	BMI: UM = 4.8% lower increase EM = used as reference IM = no significant change PM = no significant change Waist circumference: UM = 5.8% lower increase EM = used as reference IM = no significant change PM = 4% lower increase No significant difference in ATEC score or serum prolactin concentration between phenotypes	Too few PM subjects for statistical tests
Troost et al. [24]	Maximum 4.0 mg/day (< 45 kg) or 6.0 mg/day (> 45 kg) for 8 weeks	25 subjects Age range 5–15, mean age 8.6 (2.2) 23 (92%) males Diagnosed with various psychiatric disorders	UM = 2 (8%) EM = 12 (48%) IM = 6 (24%) PM = 5 (20%)	Serum risperidone concentration and risperidone/9-hydroxyrisperidone ratio Serum prolactin concentration	Serum risperidone concentration: negative correlation with number of functional CYP2D6 genes^c^ Risperidone/9-hydroxy-risperidone ratio: negative correlation with number of functional genes Serum prolactin concentration: positive correlation with number of functional genes	Too few UM subjects. Hyperprolactinemia not defined Length of time on risperidone shorter than other studies
Kohnke et al. [25]	6 mg/day for 3 months, reduced to 4 mg/day before outcomes measured	Single patient case study Age 17 Male Diagnosed with schizophrenia	PM = 1 (100%)	Serum risperidone and 9-hydroxyrisperidone concentrations. In-depth symptoms observations	Serum risperidone and 9-hydroxyrisperidone concentrations increased after 8 days of concomitant therapy of haloperidol (6 mg/day) and biperiden (2 mg/day). Patient experiences extrapyramidal symptoms while on risperidone	Single case study heightens possibility of weight/age/sex influence on results

*EM* extensive metabolizer, *IM* intermediate metabolizer, *PM* poor metabolizer, *UM* ultra-rapid metabolizer

^a^Median (interquartile range)

^b^Calarge et al. drug groups: Group 0 = no CYP2D6 inhibitors. Group 1 = weak CYP2D6 inhibitors (citalopram, escitalopram). Group 2 = intermediate CYP2D6 inhibitors (sertraline). Group 3 = strong CYP2D6 inhibitors (fluoxetine, bupropion, lamotrigine)

^c^Number of function genes increases with increased metabolic function: PM < IM < EM < UM

All studies included populations with mean or median risperidone doses that fall within the FDA effective dose range according to the FDA label (last updated 2009). Older studies generally used larger risperidone doses: for example [24, 25], included subjects using up to 6 mg/day, which is significantly greater than current recommended target dose for youth. Length of time on risperidone varied significantly between studies, from minimum 4 weeks to mean of 53.3 months.

The number of subjects per study ranged from 25 to 147, excluding [25] single patient case studies. Population size was a limiting factor for many studies, especially those that had too few subjects in the rare UM (N = 2–8) and PM (N = 1–7) metabolic phenotype groups. The combined age range for all studies was 2–21 years with mean (8.6–17.0 years) or median (7–13 years) age < 18 years for all studies. All study populations included at least 75% male subjects; this may be explained by the fact that ASD, which was included by most studies, as well as other disorders requiring risperidone are more prevalent in males [26]. Also, 80% of subjects in each study population were from a single ethnicity. This was problematic when the majority ethnicity was one in which UM and PM phenotypes are rare: for example, [18, 27] included only Thai subjects, and consequentially observed no PM phenotypes and few occurrences of UM phenotypes.

Several studies were hindered by a lack of subjects with UM and PM phenotypes. As previously mentioned, population size and ethnic composition could produce low UM and PM phenotype prevalence [16]. Another explanation for low UM and PM phenotype prevalence is that risperidone users with these phenotypes experienced poor efficacy or adverse side effects early on in treatment and subsequently discontinued therapy before the minimum length of time for inclusion was reached. This possibility is supported by a study in adults that demonstrated a significant association between PM phenotype and prompt discontinuation of risperidone use [28]. All studies except [24, 25, 29] were cross-sectional studies that only included subjects who were already taking risperidone for a minimum length of time, the shortest minimum length of time being 4 weeks by [18].

### Efficacy

Efficacy for psychotropic drugs such as risperidone is typically defined using a symptom scoring system. Only [29, 30] specifically investigated differences in efficacy between metabolic phenotypes. The former study used the Autism Treatment Evaluation Checklist (ATEC) score to evaluate risperidone efficacy. The study found no significant difference in ATEC scores between metabolic phenotypes. As [29] performed a cohort study that followed their patients from the beginning of risperidone therapy, it is unlikely that their methodology excluded patients who discontinued therapy due to poor efficacy. Youngster et al. [30] measured efficacy via a three-point scale: improvement of disruptive behaviours, no change, and worsening of disruptive behaviours, as evaluated by a neurologist. Both subjects with UM phenotype experienced no clinical response while both subjects with the PM phenotype saw improvement. It is unclear why the UM phenotype subjects continued use of risperidone for 3 months (the minimum for inclusion in this study). Further studies including more subjects with UM and PM phenotypes should be performed to investigate the relationship between efficacy and CYP2D6 metabolic phenotype.

### Pharmacokinetics

Several studies investigated differences in serum risperidone and 9-hydroxyrisperidone concentrations between CYP2D6 metabolic phenotypes, typically to validate the results of the phenotyping [18, 24, 30]. The relationship is well characterized in adults [21].

Sherwin et al. [31] investigated differences in risperidone clearance between metabolic phenotypes. Decreases in relative clearance correlated with decreases in CYP2D6 metabolic activity, though no UM phenotype subjects were included in the study. Their results are consistent with a study of risperidone clearance in adults and elders using risperidone for schizophrenia or Alzheimer’s disease [32]. Sherwin et al. [31] considered the pharmacokinetics of risperidone and 9-hydroxyrisperidone separately and suggest that differences in their pharmacokinetics could be important for occurrence of side effects. They also argued that variations in pharmacokinetics between phenotypes indicate a need for individualized dosing regimens for children within each phenotype group. Further studies should be performed to verify if such regimens are necessary.

### Hyperprolactinemia

Hyperprolactinemia is an adverse side effect of risperidone treatment. It is characterized by elevated prolactin levels which is measurable in serum. Hyperprolactinemia can lead to gynecomastia (breast growth), impotence, loss of libido, and infertility in males as well as galactorrhea (inappropriate breast milk production), amenorrhea (absence of menstruation), and sexual dysfunction in females [27].

Troost et al. [24] found a positive correlation between serum prolactin concentrations and CYP2D6 metabolic activity. They offered a biochemical explanation for this phenomenon: UM phenotype individuals have lower risperidone/9-hydroxyrisperidone ratios, and 9-hydroxyrisperidone is more polar than risperidone so it crosses the blood–brain barrier less freely. Thus, 9-hydroxyrisperidone may act more potently than risperidone on the pituitary gland (which is positioned outside of the blood–brain barrier) to induce production of prolactin [33]. While the hypothesis is intriguing, a more recent study failed to replicate its findings or expound the theory [27]. Furthermore, [24] only included two subjects with UM phenotypes and the population’s duration on risperidone was only 8 weeks. The study also did not define hyperprolactinemia nor determine if the achieved prolactin levels in any phenotype group were great enough to induce harmful side effects associated with hyperprolactinemia.

The findings of [11] were in contrast to those of [24]. The former’s study found a negative correlation between serum prolactin concentrations and CYP2D6 metabolic activity, though too few subjects with UM and PM phenotypes were available to perform statistical tests. The authors defined hyperprolactinemia: both subjects with PM phenotypes met the criteria for diagnosis while UM subjects did not. The study also included subjects who had been on risperidone for significantly longer than [24]. A duration-related effect on prolactin trends is possible. Youngster et al. [30] noted similar trends to [11]: the subjects with PM phenotypes had significantly greater serum prolactin concentrations than other phenotypes. All subjects in both UM and PM phenotypes were diagnosed with hyperprolactinemia in the [30], though no definition for hyperprolactinemia was provided. These studies did not suggest mechanisms to explain the relationship between prolactin and metabolic phenotypes. Both recommended further studies with an increased number of rarer phenotype subjects to validate their results.

Sukasem et al. [27] and dos Santos et al. [34] did not find any significant differences in prolactin concentrations or hyperprolactinemia prevalence between metabolic phenotypes, though these two studies are limited in scope by the total absence of some phenotypes. Correia et al. [29], which had a large UM phenotype population, similarly found no correlations. Thus, it is difficult to make any firm conclusions on the relationship between CYP2D6 metabolic phenotypes and prolactin. This subject remains in discussion in adult studies as well [35].

### Weight gain

Weight gain is another common side effect associated with risperidone use. The study by [29] posited that the UM metabolic phenotype is protective against risperidone-associated weight gain. Subjects with UM phenotypes experienced a 4.8% lower increase in BMI and 5.8% lower increase in waist circumference compared to the EM phenotype (note: absolute weight gain over the course of the 12-month study was approximately 10 kg per subject). The single PM phenotype subject experienced a 4% lower increase in waist circumference, but the authors claim this result should be excluded due to absence of replicates. Correia et al. [29] suggested that differences between risperidone’s and 9-hydroxyrisperidone’s affinities for receptors that regulate weight gain are responsible for the protective effects of UM phenotype. Youngster et al. [30] noted that both subjects with UM phenotypes did not report ADRs (weight gain and/or neurological extrapyramidal symptoms) while both subjects with PM phenotypes did, consistent with the theory put forward by [29] for a protective effect of UM.

### Neurological extrapyramidal symptoms

It is noteworthy that [25, 30] were the only studies to evaluate presence or absence of neurological extrapyramidal symptoms in relation to CYP2D6 metabolic phenotype. There is little data on the association between neurological extrapyramidal symptoms and metabolic phenotype, possibly because such symptoms are more noticeable and subjectively distressing than elevated prolactin and weight gain. Individuals who experience these symptoms might be more likely to discontinue risperidone treatment promptly, and thus are excluded from these studies. Some cohort studies have been done in adults but a conclusive relationship has not been elucidated [35].

### Drug interactions

Risperidone use in combination with other drugs that interact with CYP2D6 has potentially important implications when considering metabolic phenotype. A strong CYP2D6 inhibiting drug, such as fluoxetine (a selective serotonin reuptake inhibitor, SSRI) theoretically mimics the PM metabolic phenotype by reducing CYP2D6 metabolic capability [36]. These authors reported that serum concentrations of risperidone were significantly greater in subjects who were taking potent CYP2D6 inhibitor drugs, such as fluoxetine. Youngster et al. [30] and Troost et al. [24] found similar risperidone concentration results in subjects with PM phenotypes. Calarge and del Miller [36] did not perform CYP2D6 genotyping as part of their study, so it is unclear how different combinations of CYP2D6 inhibiting drugs and metabolic phenotypes would interact to affect risperidone levels or other clinical measures (prolactin, BMI, waist circumference). The study noted an effect of ethnicity that could be indicative of a concomitant drug/phenotype relationship, as prevalence of metabolic phenotypes is influenced by ethnicity. A future study that genotypes subjects who take CYP2D6 inhibiting drugs with risperidone would be informative.

Drugs that block other CYPs also affect risperidone outcomes. CYP3A4 and 3A5 enzymes also metabolize risperidone, but with a much lower activity than CYP2D6 [21]. Kohnke et al. [25] described a single PM phenotype subject who experienced a dramatic spike in serum risperidone concentration and worsening of neurological extrapyramidal symptoms after taking risperidone concomitantly with haloperidol and biperiden. The study noted that haloperidol is also metabolized by CYP3A4 and suggests that a competitive or inhibitive effect on CYP3A4 may have reduced risperidone metabolism by this enzyme. This combined with the already deficient metabolism associated with PM phenotype to elevate risperidone levels and produce side effects associated with toxicity (although haloperidol itself clearly has effects on extrapyramidal symptoms). In general, risperidone monotherapy is more common in youth, while polypharmacy is more common in adults [1, 37]. Thus, studies of concomitant drug use and metabolic phenotype may be of less frequent clinical importance in the younger age group.

## Conclusions

The results of this literature review illustrate the complex nature of pharmacogenomics and risperidone therapy. The findings reaffirm the previously characterized relationship between CYP2D6 metabolic phenotypes and risperidone/9-hydroxyrisperidone levels. The clinical importance of this relationship requires further investigation, especially to determine how changes in these levels impact drug efficacy and adverse side effects and what mechanisms underlie said impacts. In the future, researchers should strategically design studies to include more patients with UM and PM metabolic phenotypes, as these phenotypes show the most variation in treatment outcome. Overall, there may be value in CYP2D6 pharmacogenomic testing for young risperidone users, especially when treatment options are limited [4]. However, additional study is required to replicate previous findings, including in genetically different populations where less common CYP2D6 variants may be more common.

## Abbreviations

ATEC: Autism Treatment Evaluation Checklist
ASD: autism spectrum disorder
BMI: body mass index
EM: extensive metabolizer
FDA: US Food and Drug Administration
IM: intermediate metabolizer
PM: poor metabolizer
UM: ultra-rapid metabolizer
